# Cancer knowledge and health-consciousness in childhood cancer survivors following transition into adult care—results from the ACCS project

**DOI:** 10.3389/fonc.2022.946281

**Published:** 2022-09-05

**Authors:** Maria Otth, Sibylle Denzler, Tamara Diesch-Furlanetto, Katrin Scheinemann

**Affiliations:** ^1^ Division of Oncology-Hematology, Department of Pediatrics, Kantonsspital Aarau, Aarau, Switzerland; ^2^ Department of Oncology, Hematology, Immunology, Stem Cell Transplantation and Somatic Gene Therapy, University Children’s Hospital Zurich – Eleonore Foundation, Zurich, Switzerland; ^3^ Hematology and Oncology Department, Children’s Hospital of Eastern Switzerland, St. Gallen, Switzerland; ^4^ Division of Pediatric Oncology and Hematology, University Children’s Hospital Basel, Basel, Switzerland; ^5^ Department of Health Sciences and Medicine, University of Lucerne, Lucerne, Switzerland; ^6^ Department of Pediatrics, McMaster University, Hamilton, ON, Canada

**Keywords:** cancer, child, adolescent, young adult, transition, expectations, worries, self-management skills

## Abstract

**Background:**

Knowledge on chronic medical conditions in childhood cancer survivors (CCSs) is constantly growing and underlines that long-term follow-up (LTFU) care is often mandatory, also in adulthood. However, many CCSs discontinue follow-up care after transition to adult care. One reason might be that the current transition practices do not meet the needs of adolescent and young adult CCSs. We therefore aim to evaluate different transition models for Swiss CCSs by assessing their cancer knowledge, cancer worries, self-management skills, and expectations for LTFU care, following transition in two different hospital-based models.

**Methods:**

Within the Aftercare of Childhood Cancer Survivors (ACCS) study, we performed a questionnaire-based survey with a cross-sectional and longitudinal part. We included 5-year CCSs aged >16 years at recruitment who were transitioned to adult care in two hospitals between 2014 and 2021. Here, we report the results of the cross-sectional part. We compared the survivors’ cancer knowledge with medical record data and assessed cancer worries (6 questions), self-management skills (15 questions), and expectations (12 questions) by validated scales. We used descriptive statistics, chi-squared test, and t-tests to describe the results.

**Results:**

We analyzed 57 CCSs (response rate 44%), 60% of those were female, had a median age of 9 years at diagnosis and 23 years at the questionnaire. Most CCSs recalled their diagnosis (95%) and exposure to treatment modalities (98%) correctly. CCSs worried the most about potential late effects (47%) and issues with having children in the future (44%). At least 75% of CCSs agreed to 12 of the 15 self-management questions, indicating high self-management skills. The top three expectations included that physicians know the survivors’ cancer history, that visits start on time, and that physicians can always be called in case of questions.

**Conclusion:**

CCSs receiving hospital-based LTFU care have good cancer knowledge and high self-management skills. The identified worries and expectations will help to improve the LTFU care of CCSs who transition to adult care, to further inform and educate survivors and healthcare professionals about and might be relevant for other countries with a similar healthcare system.

## Introduction

Advancements in the diagnosis, treatment, and supportive care of children and adolescents diagnosed with cancer contribute to the increasing numbers of long-term childhood cancer survivors (CCSs). In parallel, knowledge on chronic medical conditions, so-called late effects, in long-term CCSs is constantly growing. Data show that the proportion of CCSs suffering from late effects increases as they get older (1–3). As a result, there is a worldwide consensus that most CCSs need long-term and often lifelong follow-up care, as most survivors experience one or more late effects due to the cancer itself or its treatment. Long-term follow-up (LTFU) care aims to reduce the burden of late effects through prevention, early detection, and treatment and to improve CCSs’ quality of life. LTFU care is performed risk-adapted and according to national or international recommendations (4–7). When CCSs reach adulthood, LTFU care should be transferred and continued in the adult setting. However, many CCSs discontinue regular follow-up care once they have left the pediatric setting, with increasing drop-off rates with longer time following treatment completion (8–10). This discrepancy between the higher prevalence of late effects in older CCSs and the decrease in adherence to LTFU care is very critical. Therefore, the transition, that is, the movement of LTFU care from the pediatric to the adult setting, is important. The transition is ideally a structured and planned process, coordinated, comprehensive, and multidisciplinary with well-informed healthcare providers. CCSs should also be well informed about their medical history and reasons for LTFU care, enabling them to navigate in the healthcare system on their own (11). The structure of the transition depends on the LTFU care model used. Dixon et al. described five models (12): 1) cancer-center model: care is provided by pediatric oncologists or dedicated survivorship care teams in the cancer center. 2) Shared-care model: care is initially provided by pediatric oncologists or survivorship care teams, which is later handed over to community healthcare providers, with specialty support provided in the cancer center. 3) Disease-specific model: care is tailored to the needs of CCSs at a particular risk, provided in a cancer center (e.g., brain tumor survivors). 4) Risk-stratified model: the individual risk of each CCS to develop late effects defines the place of LTFU care; high-risk CCSs are seen in cancer centers and low-risk CCSs in the community. 5) Consult-based model: care is delivered by community healthcare providers, including specialty support (12). None of these LTFU care models outweighs the others, and the model used depends on the local possibilities. One crucial factor, independent of the model, is education—education of the survivors and healthcare providers (11). Treatment summaries are helpful tools to educate survivors and healthcare providers and an important element of transition. The examples of these treatment summaries are the European “Survivorship Passport” (SurPass) from PanCare and the “Passport for Care” from the Children’s Oncology Group (COG) (13, 14). However, being well informed is not the only facilitating factor for a successful transition from a CCS’s perspective; further expectations concern communication, organizational aspects, or support for insurance questions (11, 15). Klassen et al. developed and validated scales to assess factors that CCSs perceive as barriers or facilitators during transition (16, 17). Their implementation has been demonstrated to be feasible in the Canadian, Japanese, and Swiss settings (18–20).

Today, we do not know which transition model fits best for Swiss CCSs and what the survivors’ cancer knowledge and expectations for LTFU care are. This multicenter, cross-sectional study aims to close this knowledge gap by assessing cancer knowledge, cancer worries, self-management, and expectations for LTFU care in CCSs following transition.

## Methods

### Study design

This study is part of the Aftercare of Childhood Cancer Survivors (ACCS) study, a prospective, multicenter, observational study, including a cross-sectional and longitudinal part (21). Three pediatric oncology centers were included, each with a different transition model. Clinic A has joint consultations with pediatric and adult oncologists/hematologists being present during the whole consultation for at least two visits. The first visit takes place in the pediatric hospital and the second in the adult hospital. Survivors decide whether further joint consultations happen before LTFU care is handed over to the adult oncologists/hematologists. Clinic B transitions CCSs to the adult clinic during one combined consultation in the pediatric hospital. In both clinics, the pediatric team is available for questions following transition. Additionally, clinic A continuously updates the survivorship care plans following transition. Clinic C transitions CCSs to the family physicians. Eligible CCSs either qualified for the longitudinal (Group 1; transition planned during next annual visit) or the cross-sectional part (Group 2, transition completed at recruitment) of the ACCS study.

### Method and data collection

Group 1 survivors received a baseline questionnaire before the annual visit and follow-up questionnaires after 3 and 15 months. Group 2 survivors received one baseline questionnaire at study inclusion (21). Here, we analyzed the cross-sectional part of the ACCS study. This includes the latest questionnaire of each CCS completed following transition, corresponding to the 15-month questionnaire of Group 1 survivors and the baseline questionnaire of Group 2 survivors. The questionnaires were identical, including sections on demographics, cancer knowledge, and validated scales to assess cancer worry, self-management, and expectations for LTFU care (**Supplemental Explanation E1**) (17). We officially translated the Cancer Worry Scale (CWS) and Self-Management Skill Scale (SMSS) into German and proved their applicability in a previous feasibility study (20). A chart review was performed by pediatric oncologists of each clinic, to collect information on diagnosis, treatment exposure, and the potential risk for late effects. Organ systems at risk were defined according to the COG LTFU guidelines V5.0 (4).

### Participants

We recruited 5-year CCSs, who have been diagnosed with cancer according to the International Childhood Cancer Classification third edition (ICCC3) and were aged <18 years at cancer diagnosis and >16 years at recruitment. CCSs further had to be ready for transition (Group 1) or have been transitioned since 2014 (Group 2). We excluded CCSs treated with surgery only, CCSs receiving cancer treatment at time of recruitment or in a palliative situation, and CCSs unable to complete the questionnaire due to cognitive disabilities or language barriers. Recruitment took place between January 2019 and March 2021.

### Ethical issues and statistical analysis

Ethics approval was granted by the cantonal ethics committee Ethikkommission Nordwest- und Zentralschweiz (EKNZ), and the study is registered at ClinicalTrials.gov (NCT04284189). We described the study population and the scales mainly descriptively. For the CWS, Klassen et al. provided a scoring system, ranging from 0 to 100, where higher numbers indicate lower degrees of worries (17). For the other scales, we combined the answer options “strongly agree” and “agree” as well as “disagree” and “strongly disagree”. We used the chi-squared test to examine differences in categorical variables and t-test for continuous variables and displayed the results graphically. Except for two questions in the “self-management skill scale”, the proportion of missing data was below 5%. For transparency, the proportion of missing data is displayed for each variable. Data were analyzed using STATA 17.0 (StataCorp. LCC, Version 17. College Station, TX, USA) and p-values <0.05 were considered statistically significant.

## Results

### Characteristics of participants

**Figure 1:**
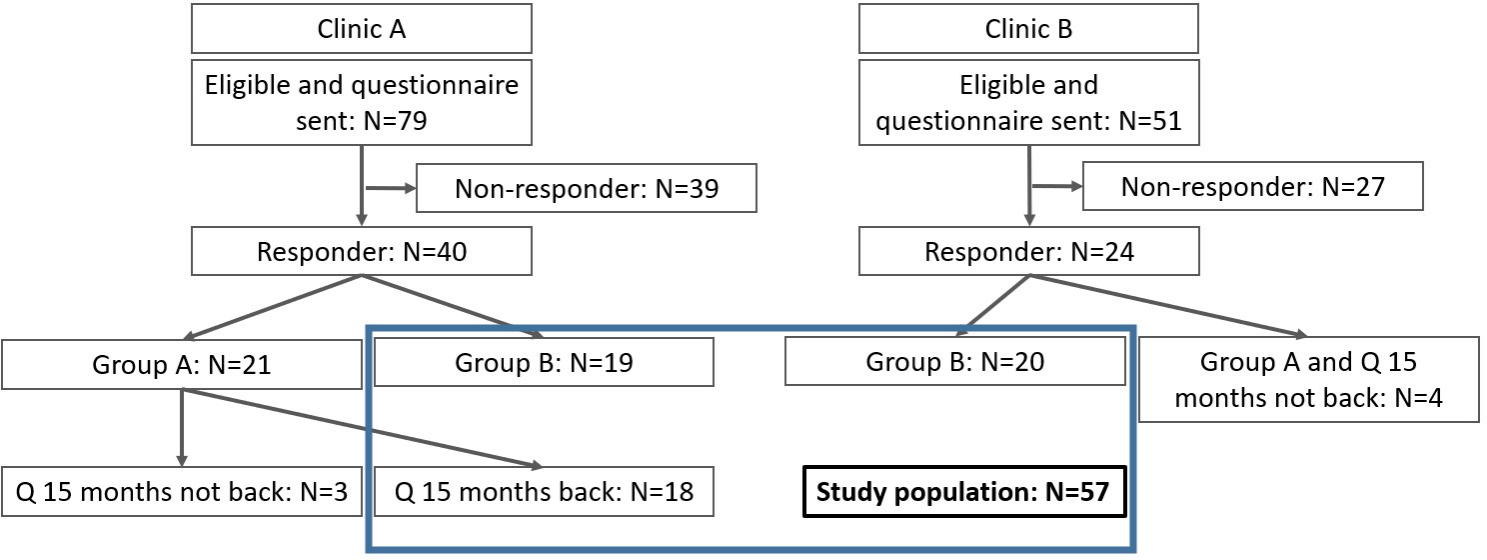
Patient Tree, n=57.

Of 130 eligible CCSs from clinic A and B, 64 (49%) participated and 57 (44%) were analyzed. Clinic C did not recruit any CCS during the study period. Seven Group 1 CCSs did not complete the 15-month questionnaire (**Figure 1**). Two-thirds of CCS (65%) were recruited by clinic A. One fourth (n=17; 28%) were Group 1 CCSs and completed the 15-month questionnaire, all recruited by clinic A. Most participants were female (60%), had a median age of 9 years (IQR 4–14) at diagnosis and 23 years (IQR 21–27) at the questionnaire (**Table 1**). Survivors from clinic A were younger at diagnosis and older at the questionnaire. The most frequent type of cancer was leukemia (37%), followed by lymphoma (16%) and tumors of the central nervous sys (9%). The distribution of primary cancer types was equal between both clinics.

**Table 1:** Patient characteristics stratified by recruiting clinic (n=57).

	Clinic A	Clinic B	Total	p-value*
**Number of participants, n (%)**	37 (65)	20 (35)	57 (100)	
**Participation in ACCS**				<0.001
- Group 2 survivors	20 (35)	20 (35)	40 (70)	
**-** Group 1 survivors	17 (30)	0	17 (30)	
**Sex**				0.968
- female	22 (39)	12 (21)	34 (60)	
- male	15 (26)	8 (14)	23 (40)	
**Age at diagnosis [years] (reported by physician)** Median (IQR)	8 (4–13) range: 1–16	14 (5–16) range: 1–19	9 (4–14) range: 1–19	0.089
**Age at survey [years]**Median (IQR)	24 (21–27) range 18–35	22 (20–25) range 18–29	23 (21–27) range: 18–35	0.108
**Current health status^1^** Median (IQR)	9 (8–10) range: 6–10	8 (7–9) range: 5–10	9 (8–9) range: 5–10	0.204
**Type of follow-up care**				0.004
- Joint clinics^2^	21	1	22 (39)	
- Adult hospital alone	11	14	25 (44)	
- Family physician and organs-specific specialists	0	1	1 (2)	
- Family physician only	2	0	2 (3)	
- Follow-up terminated	0	1	1 (2)	
- Other^3^	2	2	4 (7)	
- Missing	1	1	2 (3)	
**Type of cancer (reported by physician)**			
- Leukemia	15	6	21 (37)
- Lymphoma	7	2	9 (16)
- Tumors of the central nervous system	3	1	5 (9)
- Neuroblastoma	0	1	1 (2)
- Nephroblastoma	2	2	4 (7)	0.138
- Soft tissue sarcoma	1	3	4 (7)
- Ewing sarcoma	4	1	5 (9)
- Osteosarcoma	0	3	3 (5)
- Germ cell tumors	3	1	3 (5)
- Other types of cancer	2	0	2 (3)

*Chi-squared test for categorical variables, t-test for continuous variables; bold values represent significant p-values <0.05.

^1^Subjective assessment of current health status when answering the questionnaire, range from 0 = not at all satisfied to 10 = satisfied a lot.

^2^Joint clinics combine pediatric and adult disciplines for long-term follow-up care.

^3^Orthopedists only, moved abroad, unsure about the current situation.

### Current follow-up situation and cancer knowledge

**Figure 2:**
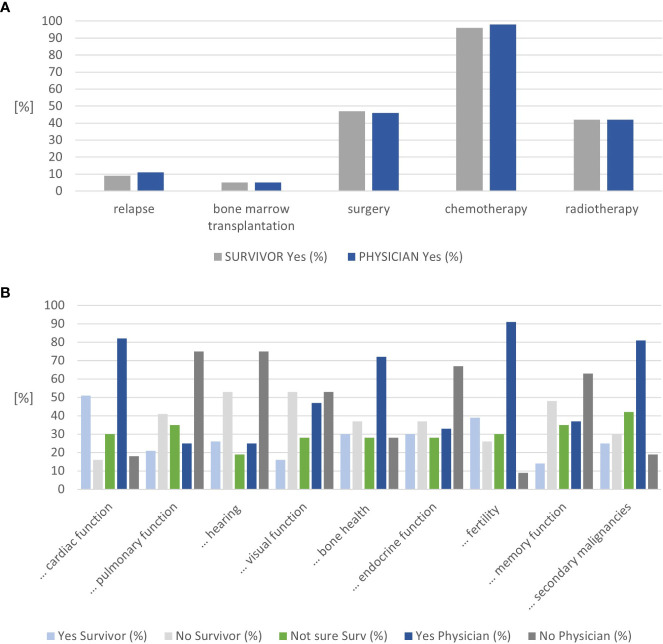
Results on cancer knowledge. **(A)** Congruence on treatment exposure mentioned by childhood cancer survivors and physicians. **(B)** Congruence on organ systems considered at risk by childhood cancer survivors and physicians (n=57).

Similar proportions of CCSs were in follow-up care by adult oncologists/hematologists alone (44%) or in joint clinics (39%). All CCSs diagnosed with leukemia, lymphoma, nephroblastoma, and osteosarcoma recalled their diagnosis correctly, representing two-thirds of all survivors (n=37, **Supplementary Table 1**). At least 90% of CCSs stated that they recall their age at diagnosis, age at treatment completion, and the cancer location. Approximately 88% of CCSs felt confident about knowing how often follow-up care visits take place and 67% on their knowledge about potential late effects (**Supplemental Table 2A**). The agreement between the survivor- and physician-reported treatment exposure was high, with 100% for radiotherapy and bone marrow transplantation and a difference of one survivor each for chemotherapy and surgery (**Figure 2A** and **Supplemental Table 2A**). This holds true for the sensitivity analysis, stratified by clinic (**Supplemental Tables 2B, C**).

The nine questions on potential late effects were answered as “not sure” by 19%–42% of CCS. Approximately 19% of CCSs were unsure about the risk for audiological late effects. In contrast, 42% of CCSs were unsure whether they were at risk for secondary malignancies or not. The organs CCSs considered themselves at risk most frequently included the heart (51%), fertility (39%), endocrine function, and bone health (30% each) (**Figure 2B** and **Supplementary Table 2A**). Physicians rated fertility (91%), heart (83%), and secondary malignancies (81%) most frequently. Combining the survivors’ answers “not sure” and “yes” resulted in an alignment between the survivors’ and physicians’ assessments for the heart, visual function, bone health, fertility, memory function, and secondary cancer (**Supplemental Figure 1A**). Combining the survivors’ answers “not sure” and “no” resulted in an alignment between the survivors’ and physicians’ assessments for lung, hearing, and endocrine function (**Supplemental Figure 1B**).

### Cancer Worry Scale

**Figure 3:**
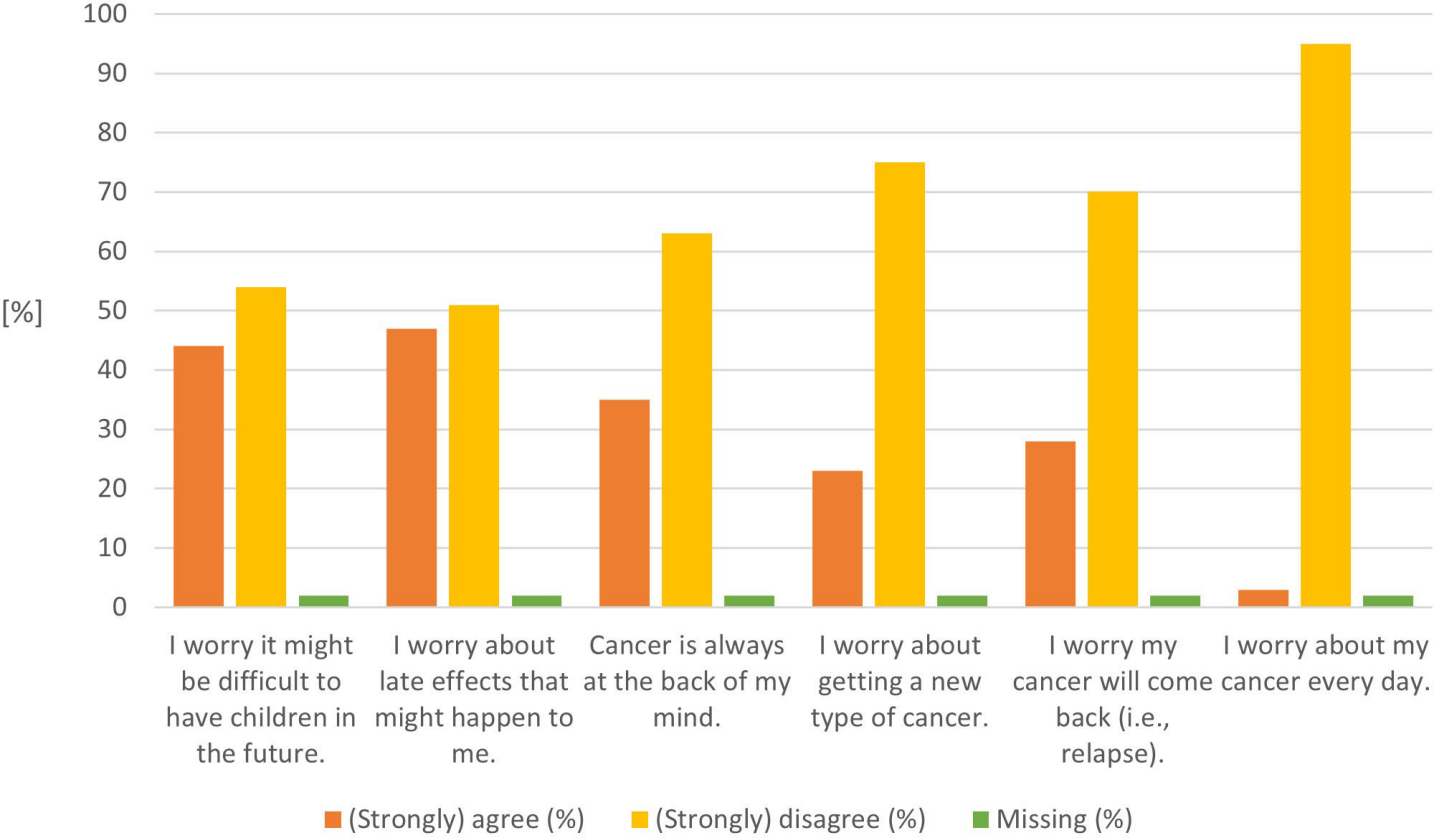
Results from the validated Cancer Worry Scale (n=57).

CCSs fear most about potential late effects (47%) and having children in the future (44%) (**Figure 3** and **Supplemental Table 3a**). One-third of CCSs have cancer always in the back of their minds (35%) or worry about cancer recurrence (28%). The mean CWS score was 62 (SD 19.88; median 60, 95%CI 51–76).

### Self-management skill-scale

At least 90% of CCSs agreed to 6 of the 15 SMSS statements (Q1–3, Q8, Q10, Q14; **Table 2**). Between 75% and 90% of CCSs agreed to further six statements (Q4, Q6, Q7, Q12, Q13, Q15). Two questions with less than 75% agreement indicate that the parents’ presence is preferred during follow-up visits (Q9, Q11).

**Table 2:** Results from the Self-Management Skills Scale, shaded areas indicating at least 75% agreement with the respective statement (n=57).

	Strongly agree n (%)	Agreen (%)	Disagreen (%)	Strongly disagreen (%)	Missingn (%)
Q1 I answer a doctor or nurse’s questions.	39 (68)	15 (26)	0	1 (2)	2 (4)
Q2 I participate in making decisions about my health.	31 (54)	22 (39)	1 (2)	0	3 (5)
Q3 I make sure I go to all my doctor’s appointments.	45 (79)	8 (14)	1 (2)	0	3 (5)
Q4 I ask the doctor or nurse questions.	17 (30)	33 (58)	5 (9)	0	2 (3)
Q5 I talk to a doctor or nurse when I have health concerns.	16 (28)	26 (46)	11 (19)	2 (3)	2 (3)
Q6 I talk about my medical conditions to people when I need to	18 (32)	27 (48)	8 (14)	2 (3)	2 (3)
Q7 I am in charge of taking any medicine that I need	37 (65)	11 (19)	3 (5)	0	6 (11)
Q8 I know how to contact a doctor if I need to.	34 (60)	19 (34)	2 (3)	0	2 (3)
Q9 I prefer it when a doctor speaks to me instead of my parent(s).	18 (32)	20 (35)	12 (21)	4 (7)	3 (5)
Q10 I can briefly describe my medical history when asked	29 (51)	22 (39)	3 (5)	0	3 (5)
Q11 I prefer to see a doctor or nurse without any parent(s) with me	21 (37)	18 (32)	11 (19)	4 (7)	3 (5)
Q12 I know how to access medical care when I travel.	21 (37)	27 (47)	6 (11)	0	3 (5)
Q13 I book my own doctor’s appointments	36 (63)	12 (21)	7 (13)	0	2 (3)
Q14 I know the type of medical insurance I have.	46 (81)	9 (16)	0	0	2 (3)
Q15 I fill my own prescriptions when I need medicine	32 (57)	16 (28)	3 (5)	2 (3)	4 (7)

### Expectation scale

Five statements were considered important by at least 75% of survivors, dealing with prepared and engaged physicians (S1, S6) and administrative issues (S2, S3, S7). Least important were statements on personal relationships with treating physicians, including the statement that visits do not always have to be with the same physician (**Table 3**). The answers to the questions Q8, Q10, and Q11 indicated that CCSs seem to have the feeling that the adult setting is more distant and less personal than in pediatric oncology.

**Table 3:** Results from the Expectation Scale, shaded areas indicating at least 75% agreement with the respective statement (n=57).

	Expectation				
When leaving the children’s hospital I expect that…	Strongly agreen (%)	Agreen (%)	Disagreen (%)	Strongly disagreen (%)	Missingn (%)
S1… my after care physician knows my cancer history.	42 (74)	13 (23)	0	0	2 (3)
S2… the visit starts on time	17 (30)	37 (65)	1 (2)	0	2 (3)
S3… I get a call when I miss an appointment	16 (28)	27 (48)	7 (12)	4 (7)	3 (5)
S4… I am always seen by the same physician	15 (27)	24 (42)	12 (21)	4 (7)	2 (3)
S5… I get a reminder before each visit	13 (23)	16 (28)	17 (30)	8 (14)	3 (5)
S6… I can always call my physician in case of questions	17 (30)	35 (62)	3 (5)	0	2 (3)
S7… other examinations for follow-up care take place on the same day	22 (39)	21 (37)	11 (19)	1 (2)	2 (3)
S8… my parents can come to the visit	6 (11)	19 (33)	15 (26)	14 (25)	3 (5)
S9… thy physician takes care of all my medical problems	14 (25)	19 (33)	17 (30)	4 (7)	3 (5)
S10 … my follow-up care physician becomes like a friend	3 (5)	11 (20)	22 (39)	19 (33)	2 (3)
S11… my follow-up care physician team spends a lot of time with me.	1 (2)	6 (11)	35 (61)	13 (23)	2 (3)
S12… I like going to my follow-up appointments	6 (11)	21 (37)	21 (37)	6 (10)	3 (5)

## Discussion

Our study shows that CCSs enrolled in hospital-based transition models were well informed about their diagnosis and treatment, which were validated by physicians’ information, with less congruence between survivors’ and physicians’ perception on organs at risk for late effects.

The survivors’ cancer knowledge is as high as in Canadian and American survivors. In a Canadian study, 93.6% of 250 survivors aged 15–26 years from three clinics recalled their diagnosis correctly (22). In an American case–control study, 98% of 87 survivors from a survivorship clinic or their parents recalled their diagnosis correctly and 90% of survivors or their parents in routine follow-up care (controls) (23). CCSs from our cohort were older at the questionnaire than the Canadian survivors (median 23 years versus median 17 years) and had a longer follow-up than the American survivors (median 13.5 years versus mean 5.2 years). CCSs from all three cohorts showed a high knowledge on treatment received, which might be explained by the structured LTFU care and the involvement of specialized healthcare professionals: transfer to specific LTFU care between 6 and 24 months from treatment completion in the Canadian cohort; evaluation in a survivorship clinic in addition to routine care in the US cohort; and transition to hospital-based joint consultations or adult oncology alone at around 18 years of age in our cohort. This highlights that different hospital-based approaches are equally good in imparting knowledge.

Swiss survivors showed moderate cancer worries, with a higher CWS score than two Canadian cohorts [mean CWS score 62 (SD 19.88) versus 50.6 (SD 18.4) and 57.8 (SD 19.4)] (18, 24). A Japanese study used the same CWS without reporting the score (19). The proportion of survivors that either agree or strongly agree to each of the six statements varies largely between the cohorts with Swiss survivors having the least cancer worries (**Supplemental Table 3B**). As our results are comparable with those from the preceding feasibility study, the lower worries are reproducible (18). Low cancer worries might be the result of well-informed survivors, including knowledge on where to go in case of symptoms or uncertainties, or not well-informed survivors, not concerned due to the lack of knowledge. As 94% of CCSs from our cohort know how to contact a doctor if they need to, we conclude that they are well informed, especially on how to navigate in the Swiss healthcare system. Including 5-year survivors only might explain the relatively low proportion of survivors worrying about cancer recurrence, as the risk of relapse is rather low in this population, again indicating well-informed survivors.

Overall, Canadian survivors showed higher self-management skills than Swiss CCSs, as a higher proportion of Canadian CCSs agreed to 11 of the 15 questions (**Supplemental Table 4)** (18). However, survivors from our cohort show higher self-management skills in administrative fields (Q13–Q15), including booking doctor’s appointments, knowing the medical insurance, or filling their own prescriptions. This might be explained by the Swiss insurance system, as adolescents must take care of their own health insurance at the age of 18. The lowest agreement between the Swiss and Canadian cohort concerns the parental involvement (Q9, Q11), where Swiss survivors favor the parental involvement. These results show that childhood cancer survivors have to learn to become independent from their parents when they grow older. They are often used to the fact that the parents take care of the appointments or answer the physicians’ questions. These answers also highlight that certain areas of long-term follow-up care have to improve to empower the survivors better.

Most factors Swiss survivors expect from their follow-up appointments are related to physicians’ knowledge about their history and structural aspects of the clinical visits. Knowing a survivors’ history is a key factor and gives confidence in the relationship between survivors and physicians (11). Having separate LTFU care consultations with dedicated physicians, experienced with possible late effects and the issues of survivors, may be beneficial. Further, LTFU care clinics often have the possibility to organize all examinations on 1 day. Starting the visit on time might also be better feasible in LTFU care clinics, separated from children undergoing active treatment. In summary, it seems feasible to implement the items considered important by the CCSs in clinic routines without much effort.

Our findings have some limitations and strengths. Absent recruitment from clinic C resulted in the analysis of two hospital-based models only, and no conclusions are possible about other transition models, especially the transition to family physicians. Clinic C was not able to identify the survivors transitioned to the family physicians between 2014 and 2021, and obviously no survivor was eligible for transition during the study period. Further, clinic B recruited Group 2 survivors only, resulting in more survivors with a longer follow-up period. Through participation bias, the results might not be representative for all Swiss CCS, either because only those with better knowledge or less late effects participated or only those with less knowledge or more late effects. As the survivors’ characteristics are comparable to other CCS cohorts, we consider the results representative for long-term CCS. The sample size made the analysis of knowledge and needs of subgroups impossible, such as separate tumor entities or treatment exposures. The participation rate was lower compared to large CCS cohorts (25–27). However, considering that some CCSs left pediatric care many years ago, the response rate of 44% is still high and comparable to other studies with the same approach (18, 19). The long follow-up period of median 14 years (IQR 11–19) is a further strength.

## Conclusion

Hospital-based follow-up care models result in high cancer knowledge and moderate cancer worries and self-management skills. Changeable structural conditions could be identified. An extension of the ACCS study is planned to evaluate the transition to family physicians.

## Data availability statement

Data can be made available upon request to the authors. Requests to access the datasets should be directed to maria.otth@ksa.ch.

## Ethics statement

This study involving human participants was reviewed and approved by the Cantonal Ethics Committee (EKNZ). Written informed consent to participate in this study was provided by the participants.

## Author contributions

Conceptualization: KS and MO; Data collection: MO, SD, and TD-F; Formal analysis: MO; Funding acquisition: KS; Writing—original draft: MO; Writing—review and editing: KS, SD, TD-F, and MO. All authors contributed to the article and approved the submitted version.

## Funding

This research was supported by the Swiss Cancer Research, grant number HSR-4359-11-2017.

## Acknowledgments

We thank all childhood cancer survivors for participating in the ACCS Project.

## Conflict of interest

The authors declare that the research was conducted in the absence of any commercial or financial relationships that could be construed as a potential conflict of interest.

## Publisher’s note

All claims expressed in this article are solely those of the authors and do not necessarily represent those of their affiliated organizations, or those of the publisher, the editors and the reviewers. Any product that may be evaluated in this article, or claim that may be made by its manufacturer, is not guaranteed or endorsed by the publisher.
