# Supplementary material for: Cancer knowledge and health-consciousness in childhood cancer survivors following transition into adult care—results from the ACCS project
Source: Front Oncol. 2022 Sep 5;12:946281. doi: 10.3389/fonc.2022.946281 (PMC9483214; doi:10.3389/fonc.2022.946281)
Supplement: Supplementary file 1 [file DataSheet_1.docx]

Supplementary Material

**Cancer Knowledge and Health-Consciousness in Childhood Cancer Survivors following Transition into Adult Care – First results from the ACCS Project**

Maria Otth, Sibylle Denzler, Tamara Diesch-Furlanetto, Katrin Scheinemann

**Supplementary material**

**Supplemental Explanation E1:** **Content of the questionnaires used**

**General Information**

1. Your current age in years ______________
2. Your sex (male, female)
3. How do you rate your current health status on a scale from 0 (=not at all satisfied) to 10 (=satisfied a lot)

**Diagnosis and Treatment** *(Answer options: “yes”, “no”, “not sure”)*

1. I know which type of cancer I had
2. I know how old I was at diagnosis
3. I know how old I was at treatment completion
4. I know where in my body the cancer was located
5. I know some or all late effects, that might be cause by the cancer treatment I received
6. I know how frequent I have to come to follow-up care
7. I had at least one cancer relapse
8. I had a bone marrow transplantation
9. I had surgery to remove the cancer
10. I was treated with chemotherapy
11. I was treated with radiotherapy
12. I know the names of some or all chemotherapeutic agents I received and that might cause late effects *(only applicable or those who answered to Q10 with “yes”)*
13. I know where in my body I received radiotherapy *(only applicable or those who answered to Q11 with “yes”)*
14. Type of cancer *(tick boxes)*:
    1. Leukemia
    2. Tumor of the central nervous system
    3. Lymphoma
    4. Neuroblastoma
    5. Nephorblastoma
    6. Germ cell tumor
    7. Soft tissue sarcoma
    8. Ewing sarcoma
    9. Osteosarcoma
    10. Other
15. Age at cancer diagnosis in years ___________
16. Due to the treatment received I have an increased risk to suffer from late effects of… *(Answer options: “yes”, “no”, “not sure”)*
    1. …the heart
    2. …the lungs
    3. …the ears (e.g. hearing impairment)
    4. …the eyes (e.g. cataract)
    5. …the bones (e.g. osteoporosis)
    6. …the hormonal system
    7. …to have children (fertility)
    8. …the memory function
    9. …secondary cancer
    10. … other ____________

**Cancer Worry Scale**

**From**: Klassen AF, Grant C, Barr R, Brill H, Kraus de Camargo O, Ronen GM, et al. Development and validation of a generic scale for use in transition programmes to measure self-management skills in adolescents with chronic health conditions: the TRANSITION-Q. Child: care, health and development. 2015;41(4):547-58

**Self- Management Skills Scale**

**From**: Klassen AF, Grant C, Barr R, Brill H, Kraus de Camargo O, Ronen GM, et al. Development and validation of a generic scale for use in transition programmes to measure self-management skills in adolescents with chronic health conditions: the TRANSITION-Q. Child: care, health and development. 2015;41(4):547-58

**Adapted Expectation Scale**

**From**: Klassen AF, Grant C, Barr R, Brill H, Kraus de Camargo O, Ronen GM, et al. Development and validation of a generic scale for use in transition programmes to measure self-management skills in adolescents with chronic health conditions: the TRANSITION-Q. Child: care, health and development. 2015;41(4):547-58

**Supplementary Table 1:** Congruence of the mentioned tumor type of childhood cancer survivors and physicians (n=57)

|  |  | **Tumor type by physicians** | | | | | | | | | |  |
| --- | --- | --- | --- | --- | --- | --- | --- | --- | --- | --- | --- | --- |
|  |  | Leukemia | Neuroblastoma | Soft Tissue Sarcoma | CNS Tumors | Nephroblastoma | Ewing Sarcoma | Lymphoma | Germ Cell Tumors | Osteosarcoma | Other | **Total** |
| **Tumor type by survivor** | Leukemia | 21 | 0 | 0 | 0 | 0 | 0 | 0 | 0 | 0 | 0 | **21** |
|  | Neuroblastoma | 0 | 1 | 0 | 1 | 0 | 0 | 0 | 0 | 0 | 0 | **2** |
|  | Soft Tissue Sarcoma | 0 | 0 | 3 | 0 | 0 | 0 | 0 | 0 | 0 | 1 | **4** |
|  | CNS Tumors | 0 | 0 | 0 | 3 | 0 | 0 | 0 | 0 | 0 | 0 | **3** |
|  | Nephroblastoma | 0 | 0 | 0 | 0 | 4 | 0 | 0 | 0 | 0 | 0 | **4** |
|  | Ewing Sarcoma | 0 | 0 | 1 | 0 | 0 | 4 | 0 | 0 | 0 | 0 | **5** |
|  | Lymphoma | 0 | 0 | 0 | 0 | 0 | 0 | 9 | 0 | 0 | 0 | **9** |
|  | Germ Cell Tumors | 0 | 0 | 0 | 1* | 0 | 0 | 0 | 2 | 0 | 0 | **3** |
|  | Osteosarcoma | 0 | 0 | 0 | 0 | 0 | 0 | 0 | 0 | 3 | 0 | **3** |
|  | Other | 0 | 0 | 0 | 0 | 0 | 0 | 0 | 0 | 0 | 1 | **1** |
|  | Missing | 0 | 0 | 0 | 0 | 0 | 1 | 0 | 1 | 0 | 0 | **2** |
|  | **Total** | **21** | **1** | **4** | **5** | **4** | **5** | **9** | **3** | **3** | **2** | **57** |

*Germ cell tumor of the central nervous system (CNS)

**Supplemental Table 2a)** Cancer knowledge, knowledge on potential late effects and comparison with physician reported exposure and risk (n=57)

|  | **Survivor** | | | | **Physician** | |
| --- | --- | --- | --- | --- | --- | --- |
|  | Yes  n (%) | No  n (%) | Not sure  n (%) | Missing  n (%) | **Yes**  **n (%)** | **No**  **n (%)** |
| I know which type of cancer I had | 52 (91) | 0 | 4 (7) | 1 (2) |  |  |
| I know how old I was at diagnosis | 56 (98) | 0 | 0 | 1 (2) |  |  |
| I know how old I was at completion of treatment | 51 (90) | 0 | 4 (7) | 2 (3) |  |  |
| I know where the cancer was located in my body | 53 (93) | 1 (2) | 2 (3) | 1 (2) |  |  |
| I know some or all late effects that may be cause by the treatment I received | 38 (67) | 7 (12) | 11 (19) | 1 (2) |  |  |
| I know how often I have to go to follow-up visits | 50 (88) | 2 (3) | 4 (7) | 1 (2) |  |  |
| I had at least one relapse of my disease | 5 (9) | 49 (86) | 1 (2) | 2 (3) | 6 (11) | 51 (89) |
| I had a bone marrow transplantation | 3 (5) | 46 (81) | 6 (11) | 2 (3) | 3 (5) | 54 (95) |
| I had a surgery to treat my cancer | 27 (47) | 27 (47) | 2 (3) | 1 (2) | 26 (46) | 31 (54) |
| I was treated with chemotherapy | 55 (96) | 1(2) | 0 | 1 (2) | 56 (98) | 1 (2) |
| I was treated with radiotherapy | 24 (42) | 29 (51) | 2 (3) | 2 (3) | 24 (42) | 3 (58) |
| I know the names of some or all chemotherapeutic agents I was treated with *(apply only to those with chemotherapy; n=55)* | 11 (20) | 26 (48) | 15 (27) | 3 (5) |  |  |
| I know which parts of my body were irradiated *(apply only to those with radiotherapy; n=24)* | 20 (83) | 0 | 3 (13) | 1 (4) |  |  |
| I have an increased risk to suffer from late effects that impair … |  |  |  |  |  |  |
| … cardiac function | 29 (51) | 9 (16) | 17 (30) | 2 (3) | 47 (83) | 10 (17) |
| … pulmonary function | 12 (21) | 23 (41) | 20 (35) | 2 (3) | 14 (25) | 43 (75) |
| … hearing | 15 (26) | 30 (53) | 11 (19) | 1 (2) | 14 (25) | 43 (75) |
| … visual function | 9 (16) | 30 (53) | 16 (28) | 2 (3) | 27 (47) | 30 (53) |
| … bone health | 17 (30) | 21 (37) | 16 (28) | 3 (5) | 41 (72) | 16 (28) |
| … endocrine function | 17 (30) | 21 (37) | 16 (28) | 3 (5) | 19 (33) | 38 (67) |
| … fertility | 22 (39) | 15 (26) | 17 (30) | 3 (5) | 52 (91) | 5 (9) |
| … memory function | 8 (14) | 27 (48) | 20 (35) | 2 (3) | 21 (37) | 36 (63) |
| … cause secondary malignancies | 14 (25) | 17 (30) | 24 (42) | 2 (3) | 46 (81) | 11 (19) |
| … other function(s) |  |  |  |  |  |  |

**Supplemental Table 2b)** Cancer knowledge, knowledge on potential late effects and comparison with physician reported exposure and risk in survivors from Clinic A (n=37)

|  | **Survivor** | | | | **Physician** | |
| --- | --- | --- | --- | --- | --- | --- |
|  | Yes  n (%) | No  n (%) | Not sure  n (%) | Missing  n (%) | **Yes**  **n (%)** | **No**  **n (%)** |
| I had at least one relapse of my disease | 3 (8) | 32 (86) | 1 (3) | 1 (3) | 3 (8) | 34 (92) |
| I had a bone marrow transplantation | 2 (5) | 29 (78) | 5 (13) | 1 (3) | 3 (8) | 34 (92) |
| I had a surgery to treat my cancer | 15 (41) | 20 (54) | 2 (5) | 0 | 14 (38) | 23 (62) |
| I was treated with chemotherapy | 36 (97) | 1 (3) | 0 | 0 | 36 (97) | 1 (3) |
| I was treated with radiotherapy | 17 (46) | 17 (46) | 2 (5) | 1 (3) | 17 (46) | 20 (54) |
| I have an increased risk to suffer from late effects that impair … |  |  |  |  |  |  |
| … cardiac function | 19 (51) | 5 (14) | 12 (32) | 1 (3) | 29 (78) | 8 (22) |
| … pulmonary function | 9 (24) | 15 (40) | 13 (35) | 0 | 9 (24) | 28 (76) |
| … hearing | 8 (22) | 20 (54) | 9 (24) | 0 | 8 (22) | 29 (78) |
| … visual function | 7 (19) | 20 (54) | 19 (27) | 0 | 24 (65) | 13 (35) |
| … bone health | 14 (38) | 12 (32) | 10 (27) | 1 (3) | 27 (73) | 10 (27) |
| … endocrine function | 12 (32) | 13 (36) | 12 (32) | 0 | 13 (35) | 24 (65) |
| … fertility | 15 (40) | 11 (30) | 10 (27) | 1 (3) | 34 (92) | 3 (8) |
| … memory function | 6 (16) | 18 (49) | 13 (35) | 0 | 14 (38) | 23 (62) |
| … cause secondary malignancies | 9 (24) | 11 (30) | 17 (46) | 0 | 36 (97) | 1 (3) |

**Supplemental Table 2c)** Cancer knowledge, knowledge on potential late effects and comparison with physician reported exposure and risk in survivors from Clinic B (n=20)

|  | **Survivor** | | | | **Physician** | |
| --- | --- | --- | --- | --- | --- | --- |
|  | Yes  n (%) | No  n (%) | Not sure  n (%) | Missing  n (%) | **Yes**  n (%) | **No**  n (%) |
| I had at least one relapse of my disease | 2 (10) | 17 (85) | 0 | 1 (5) | 3 (15) | 17 (85) |
| I had a bone marrow transplantation | 1 (5) | 17 (85) | 1 (5) | 1 (5) | 0 | 20 (100) |
| I had a surgery to treat my cancer | 12 (60) | 7 (35) | 0 | 1 (5) | 12 (60) | 8 (40) |
| I was treated with chemotherapy | 19 (95) | 0 | 0 | 1 (5) | 20 (100) | 0 |
| I was treated with radiotherapy | 7 (35) | 12 (60) | 0 | 1 (5) | 7 (35) | 13 (65) |
| I have an increased risk to suffer from late effects that impair … |  |  |  |  |  |  |
| … cardiac function | 10 (50) | 4 (20) | 5 (25) | 1 (5) | 18 (90) | 2 (10) |
| … pulmonary function | 3 (15) | 8 (40) | 7 (35) | 2 (10) | 5 (25) | 15 (75) |
| … hearing | 7 (35) | 10 (50) | 2 (10) | 1 (5) | 6 (30) | 14 (70) |
| … visual function | 2 (10) | 10 (50) | 6 (30) | 2 (10) | 3 (15) | 17 (85) |
| … bone health | 3 (15) | 9 (45) | 6 (30) | 2 (10) | 14 (70) | 6 (30) |
| … endocrine function | 5 (25) | 8 (40) | 4 (20) | 3 (15) | 6 (30) | 14 (70) |
| … fertility | 7 (35) | 4 (20) | 7 (35) | 2 (10) | 18 (90) | 2 (10) |
| … memory function | 2 (10) | 9 (45) | 7 (35) | 2 (10) | 7 (35) | 13 (65) |
| … cause secondary malignancies | 5 (25) | 6 (30) | 7 (35) | 2 (10) | 10 (50) | 10 (50) |

**Supplemental Table 3a**: Results from the Cancer Worry Scale from the Swiss Cohort, n=57

|  | **Strongly agree**  **n (%)** | **Agree**  **n (%)** | **Disagree**  **n (%)** | **Strongly disagree**  **n (%)** | **Missing**  **n (%)** |
| --- | --- | --- | --- | --- | --- |
| I worry it might be difficult to have children in the future. | 13 (23) | 12 (21) | 19 (33) | 12 (21) | 1 (2) |
| I worry about late effects that might happen to me. | 5 (9) | 22 (38) | 18 (32) | 11 (19) | 1 (2) |
| Cancer is always at the back of my mind. | 4 (7) | 16 (28) | 22 (38) | 14 (25) | 1 (2) |
| I worry about getting a new type of cancer. | 2 (4) | 11 (19) | 19 (33) | 24 (42) | 1 (2) |
| I worry my cancer will come back (i.e., relapse). | 1 (2) | 15 (26) | 20 (35) | 20 (35) | 1 (2) |
| I worry about my cancer every day. | 0 | 2 (3) | 16 (28) | 38 (67) | 1 (2) |

**Supplemental Table 3b**: Comparison of the results from the Cancer Worry Scale

|  | **Swiss cohort (current)** | **Swiss cohort (feasibility study)^a^** | **Kam et al (Canada) ^b^** | **Wang et al (Canada) ^c^** | **Ishida et al (Japan) ^d^** |
| --- | --- | --- | --- | --- | --- |
|  | **Strongly agree or agree** | **Strongly agree or agree** | **Strongly agree or agree** | **Strongly agree or agree** | **Strongly agree or agree** |
| I worry it might be difficult to have children in the future. | 44% | 40% | 60% | 46% | ~51%* |
| I worry about late effects that might happen to me. | 47% | 44% | 75% | 54% | ~46%* |
| Cancer is always at the back of my mind. | 35% | 42% | 57% | 44% | ~58%* |
| I worry about getting a new type of cancer. | 23% | 24% | 54% | 37% | ~51%* |
| I worry my cancer will come back (i.e., relapse). | 28% | 26% | 42% | 38% | ~61%* |
| I worry about my cancer every day. | 3% | 2% | 14% | 14% | ~9%* |
| **Number of participants** | 57 | 50 | 73 | 250 | 242 |
| **Response rate (included in analysis)** | 44% | na | 40% | 75.5% | 42.5% |

*estimated number as result only available from a figure

^a^ Otth M, Wechsler P, Denzler S, Koehler H, Scheinemann K. Determining transition readiness in Swiss childhood cancer survivors – a feasibility study. BMC Cancer. 2021;21(1):84

^b^ Kam V, Hendershot E, Anderson L, Marjerrison S. Evaluation of a joint adult and pediatric clinic for cancer survivorship care. Pediatric blood & cancer. 2017;64(9)

^c^ Wang R, Syed IA, Nathan PC, Barr RD, Rosenberg-Yunger ZR, Klassen AF. Exploring Cancer Worry in Adolescent and Young Adult Survivors of Childhood Cancers. Journal of adolescent and young adult oncology. 2015;4(4):192-9.

^d^ Ishida Y, Tezuka M, Hayashi M, Inoue F. Japanese childhood cancer survivors' readiness for care as adults: a cross-sectional survey using the Transition Scales. Psycho-oncology. 2017;26(7):1019-26

**Supplemental Table 4**: Comparison of the results from the Self-Management Skill Scale

|  | **Swiss cohort (current)** | **Swiss cohort (feasibility study)^a^** | **Kam et al (Canada) ^b^** | **Ishida et al (Japan) ^c^*** |
| --- | --- | --- | --- | --- |
|  | **Strongly agree or agree (%)** | **Strongly agree or agree (%)** | **Strongly agree or agree (%)** | **Strongly agree or agree (%)** |
| Q1 I answer a doctor or nurse’s questions. | 94 | 100 | 100 | ~94 |
| Q2 I participate in making decisions about my health. | 93 | 100 | 100 | ~89 |
| Q3 I make sure I go to all my doctor’s appointments. | 93 | 98 | 100 | ~91 |
| Q4 I ask the doctor or nurse questions. | 88 | 84 | 96 | ~85 |
| Q5 I talk to a doctor or nurse when I have health concerns. | 74 | 72 | 96 | ~90 |
| Q6 I talk about my medical conditions to people when I need to | 80 | 84 | 95 | ~90 |
| Q7 I am in charge of taking any medicine that I need | 84 | 94 | 99 | ~95 |
| Q8 I know how to contact a doctor if I need to. | 94 | 98 | 95 | ~85 |
| Q9 I prefer it when a doctor speaks to me instead of my parent(s). | 67 | 62 | 89 | ~81 |
| Q10 I can briefly describe my medical history when asked | 90 | 90 | 95 | ~89 |
| Q11 I prefer to see a doctor or nurse without any parent(s) with me | 69 | 74 | 70 | ~26 |
| Q12 I know how to access medical care when I travel. | 84 | 94 | 65 | ~59 |
| Q13 I book my own doctor’s appointments | 84 | 80 | 73 | ~81 |
| Q14 I know the type of medical insurance I have. | 97 | 86 | 63 | ~66 |
| Q15 I fill my own prescriptions when I need medicine | 85 | 82 | 80 | ~73 |

*estimated number as result only available from a figure

^a^ Otth M, Wechsler P, Denzler S, Koehler H, Scheinemann K. Determining transition readiness in Swiss childhood cancer survivors – a feasibility study. BMC Cancer. 2021;21(1):84

^b^ Kam V, Hendershot E, Anderson L, Marjerrison S. Evaluation of a joint adult and pediatric clinic for cancer survivorship care. Pediatric blood & cancer. 2017;64(9)

^c^ Ishida Y, Tezuka M, Hayashi M, Inoue F. Japanese childhood cancer survivors' readiness for care as adults: a cross-sectional survey using the Transition Scales. Psycho-oncology. 2017;26(7):1019-26

**Supplemental Figure 1:** Congruence on organ systems considered at risk by childhood cancer survivors and physicians when those considered “unsure” by survivors were assigned to the a) “yes” category or b) “no” category (n=57)

a

b

Legend: In Figure 1a survivors’ answers “yes” and “not sure” are combined in “Yes combined Survivors”. In Figure 1b survivors’ answers “no” and “not sure” are combined in “No combined Survivors”.
